# Epigenetic and Inflammatory Signatures in Familial Mediterranean Fever: Implication of miR-204-3p and miR-223-3p in Pyrin-Mediated Immune Regulation

**DOI:** 10.3390/jcm15062107

**Published:** 2026-03-10

**Authors:** Ramila Hajiyeva, Sinem Durmus, Ufuk Cakatay, Kaan Can Demirbas, Sezgin Sahin, Amra Adrovic, Remise Gelisgen, Ozgur Kasapcopur, Hafize Uzun

**Affiliations:** 1 Department of Medical Biochemistry, Cerrahpasa Medical School, Istanbul University-Cerrahpasa, 34098 Istanbul, Türkiye; ramila.hajiyeva@gmail.com (R.H.); cakatay@yahoo.com (U.C.); remisagelisgen@hotmail.com (R.G.); 2 Department of Medical Biochemistry, Faculty of Medicine, İzmir Kâtip Çelebi University, 35620 Izmir, Türkiye; durmus.sinem@gmail.com; 3 Department of Pediatric Rheumatology, Cerrahpasa Medical School, Istanbul University-Cerrahpasa, 34098 Istanbul, Türkiye; demirbaskaancan@gmail.com (K.C.D.); sezgin@istanbul.edu.tr (S.S.); ozgurkasapcopur@hotmail.com (O.K.); 4 Department of Pediatric Rheumatology, Faculty of Medicine, Koc University, 34450 Istanbul, Türkiye; amra.adrovic@istanbul.edu.tr; 5 Department of Medical Biochemistry, Faculty of Medicine, İstanbul Atlas University, 34408 Istanbul, Türkiye

**Keywords:** cytotoxic T-lymphocyte antigen 4, deltex1, familial Mediterranean fever, inflammation, miR-204-3p, miR-223-3p

## Abstract

**Objectives:** Familial Mediterranean fever (FMF) is an autoinflammatory disease caused by *MEFV* mutations, leading to recurrent fever and inflammation. Dysregulation of innate and adaptive immunity, including altered expression of microRNAs and immune regulatory molecules, may contribute to disease heterogeneity. The role of CTLA-4, DTX1, and selected miRNAs in FMF pathogenesis remains unclear. **Methods**: We conducted a case–control study including 48 pediatric FMF patients and 36 age- and sex-matched healthy controls. Serum miR-204-3p and miR-223-3p levels were assessed via qRT-PCR. Plasma concentrations of pyrin, CTLA-4, and DTX1 were measured using ELISA. Clinical data and MEFV mutation types were analyzed in relation to biomarker levels. **Results:** There was no statistical significance between the groups in plasma CTLA-4 levels. Serum miR-204-3p, miR-223-3p, and plasma DTX1 levels were found to be significantly lower in FMF patients, while plasma pyrin levels (*p* < 0.05, in all) were significantly higher. CTLA-4 levels were positively correlated with pyrin and DTX1 levels (r = 0.602; *p* < 0.001; r = 0.740; *p* < 0.001, respectively). **Conclusions:** miR-204-3p and miR-223-3p may be associated with FMF pathogenesis. Increased levels of the pyrin protein, encoded by the *MEFV* gene, may have an important role in apoptotic and inflammatory signaling pathways. A decrease in DTX1 levels and a positive correlation between DTX1 and CTLA-4 suggest that subclinical inflammation may continue in attack-free periods in FMF patients.

## 1. Introduction

Familial Mediterranean fever (FMF) is a monogenic *autoinflammatory* disorder predominantly affecting populations of Mediterranean ancestry. It is characterized by recurrent episodes of fever and serositis, often beginning in childhood. Recent improvements in diagnosis and treatment protocols have made it possible to control the long-term complications of the disease, including AA amyloidosis, to an extent that the complication rates and the mortality associated with it are comparable to those observed in age-matched healthy children. However, constant attacks and the physical symptoms of this disease are still associated with decreased quality of life scores and educational performance. Furthermore, there is strong heterogeneity in the phenotype of the disease in terms of attack frequency and severity of symptoms and triggers [1,2].

FMF is caused by mutations in the *MEFV* gene, which encodes the pyrin protein. Pyrin plays a crucial role in modulating inflammasome activation and interleukin-1β (IL-1β) production in response to danger signals. Dysfunctional pyrin, due to *MEFV* mutations, results in uncontrolled innate immune activation, which underlies the pathogenesis of FMF. Although FMF is classically viewed as a disease of innate immunity, some descriptions of the autoinflammatory disease involve an association with activation of the adaptive immune system and potentially immune dysfunctions, such as susceptibility to infections, autoimmunity, or uncontrolled hyperinflammation [3,4].

MicroRNAs (miRNAs) are small, non-coding RNA molecules that regulate gene expression post-transcriptionally and have been implicated in various autoinflammatory and autoimmune conditions. In FMF, several miRNAs have been found to exhibit dysregulated expression, reflecting persistent subclinical inflammation even during attack-free periods. Notably, miR-107, let-7d-5p, miR-148b-3p, and miR-223-3p have been shown to be downregulated, whereas miR-21-5p and miR-144-3p are typically upregulated [5,6]. Additionally, the levels of miR-204-3p have been found to decrease in serum during attack periods, reflecting an increase in inflammatory cytokines [7].

Cytotoxic T-lymphocyte antigen-4 (CTLA-4) is an immune regulatory receptor that inhibits T cell activation by competing with CD28 for binding to CD80/86 on antigen-presenting cells. DTX1, an E3 ubiquitin ligase, is involved in Notch signaling and has been shown to negatively regulate T cell responses in murine models. Both these molecules have major roles in immune regulation, and their defects have been shown in autoimmune conditions. Currently, there is no study on the levels of CTLA-4 and DTX1, the primary markers of the adaptive immune system, in FMF patients [8].

Although the clinical and genetic definition of FMF is widely known, the current literature lacks sufficient data on attack control and the role of environmental and epigenetic factors on different phenotypes of patients with known mutations. Investigation of epigenetic factors, especially the role of miRNAs, can shed light on how the disease progresses continuously. Therefore, in this study, we aimed to investigate the serum expression levels of miR-223-3p and miR-204-3p, as well as the plasma concentrations of pyrin, CTLA-4, and DTX1, in pediatric FMF patients with different mutation types. We hypothesize that these molecules are differentially regulated in patients with different genotypes and may serve as noninvasive biomarkers that might reflect the progression of the disease and its phenotype.

## 2. Materials and Methods

### 2.1. Study Design and Participants

This was an observational case–control study that included 48 pediatric patients with a confirmed diagnosis of FMF and 36 age- and sex-matched healthy controls. All participants were recruited from the Pediatric Rheumatology Department of Istanbul University-Cerrahpasa, Cerrahpasa Medical Faculty. FMF diagnosis was made according to the established criteria in a Turkish cohort in 2009 [9]. Thirty completely healthy children of the same age group admitted to the Pediatric Outpatient Clinic for a variety of reasons were included as the control group.

All patient data, including demographic characteristics, clinical history, family history, mutation analysis, treatment regimens, and laboratory parameters, were collected through a structured data collection form and verified against medical records.

### 2.2. Inclusion and Exclusion Criteria

#### 2.2.1. Inclusion Criteria:

- Age < 18 years;
- Confirmed FMF diagnosis as per the mentioned criteria;
- Consent from legal guardians.

#### 2.2.2. Exclusion Criteria:

- Age ≥ 18 years;
- Concomitant inflammatory, autoimmune, or malignant disease;
- Chronic hepatic or renal insufficiency;
- Neurological or severe psychiatric disorders;
- Diagnosis of amyloidosis;
- Failure to provide informed consent;
- Having an attack for the last four weeks.

Control subjects were healthy children of similar age with no known systemic or inflammatory illness, presenting for routine follow-up at the pediatric outpatient clinic.

### 2.3. Ethical Approval

The study was approved by the Ethics Committee of Cerrahpasa Medical Faculty, Istanbul University-Cerrahpasa (Approval Date: 20 March 2019; File No: 83045809-604.01.02). All participants’ legal guardians provided written informed consent. The study was conducted in accordance with the Declaration of Helsinki.

### 2.4. Sample Collection and Processing

Venous blood samples were collected from patients and controls in the morning following an overnight fast of 10–12 h. Blood was drawn into EDTA-coated tubes (for plasma and RNA extraction) and plain serum tubes (for serum collection). Samples were immediately centrifuged at 4000 rpm for 10 min at 4 °C. The resulting serum and plasma were aliquoted and stored at −80 °C until analysis.

### 2.5. Biomarker Measurements

#### 2.5.1. Pyrin, CTLA-4, and DTX1 Levels

Plasma levels of pyrin, CTLA-4, and Deltex-1 (DTX1) were measured using commercial ELISA kits (Bioassay Technology Laboratory, Shanghai, China) according to the manufacturer’s protocols (Pyrin: Cat. No. E6050Hu; CTLA-4: Cat. No. E0277Hu; DTX1: Cat. No. E77404Hu). All assays were performed in duplicate. Intra- and inter-assay coefficients of variation (CV%) were 8% and 10%, respectively, for each marker.

#### 2.5.2. MicroRNA Analysis

##### RNA Extraction and cDNA Synthesis

Total miRNA was extracted from serum samples using the Extract ME miRNA Kit (Suarge Biotechnology, Istanbul, Türkiye), following the manufacturer’s instructions. The RNA concentration and purity were evaluated spectrophotometrically (Nanodrop). Complementary DNA (cDNA) was synthesized from total RNA using the WizScript High-Capacity cDNA Synthesis Kit (Cat. No. W2211).

##### Quantitative Real-Time PCR (qRT-PCR)

qRT-PCR was conducted using the Applied Biosystems StepOnePlus™ System. The expression levels of miR-204-3p, miR-223-3p, and the internal control snRNA-RNU6 were measured using sequence-specific primers provided by Suarge Biotechnology (Catalog Nos: SUA-MIREXs, MIREXs-U6). All reactions were performed in triplicate. Relative expression was calculated using the 2^−ΔΔCT^ method. ΔCT values were used for group comparisons. RNU6 served as the endogenous normalization control.

#### 2.5.3. Mutation Analysis

MEFV mutation analysis was performed using fragment analysis on the ABI PRISM^®^ 3500 Genetic Analyzer (Applied Biosystems, Carlsbad, CA, USA). Mutation types and zygosity were recorded for each patient. MEFV mutation data were obtained from patients’ clinical genetic records. Genotyping was performed in routine clinical diagnostic laboratories using targeted mutation panels covering the most common MEFV variants associated with FMF. Full gene sequencing was not performed in all patients. Variant classification was conducted at the individual variant level, and pathogenicity assessment was based on curated mutation databases and published literature. Zygosity status (heterozygous, homozygous, or compound heterozygous) was recorded for all detected variants.

R202Q was classified as a variant of uncertain significance or low penetrance and was not considered an independent disease-causing mutation. When present together with established pathogenic exon 10 mutations, patients were categorized according to exon 10 involvement.

#### 2.5.4. Statistical Analysis

Statistical analyses were performed using SPSS version 26.0 (IBM Corp., Armonk, NY, USA). Descriptive statistics are reported as mean ± standard deviation (SD), median (minimum–maximum), or frequency (percentage), as appropriate. Normality of distribution was assessed using the Shapiro–Wilk test only for continuous variables, including serum biomarker levels (pyrin, CTLA-4, DTX1) and quantitative miRNA expression values. For comparisons between two independent groups, the independent-sample *t*-test was used for normally distributed variables, while the Mann–Whitney U test was applied for non-normally distributed data. For comparisons involving more than two groups, one-way analysis of variance (ANOVA) was used for normally distributed variables, followed by Bonferroni post-hoc correction for multiple comparisons. When normality assumptions were not met, appropriate non-parametric alternatives were applied. Categorical variables, including MEFV mutation status, genotype groupings, and other frequency-based data, were analyzed using chi-square tests. miRNA expression levels were analyzed using ΔCT values for group comparisons, and relative expression (fold change) was calculated using the 2^−ΔΔCT^ method. Associations between quantitative variables were evaluated using Spearman correlation analysis. Statistical significance was defined as *p* < 0.05.

## 3. Results

### 3.1. Clinical and Basic Laboratory Findings of Subjects

The study included 48 (22 males, 26 females) patients with FMF and 36 (23 males, 13 females) age- and sex-matched healthy controls. The mean age of onset and age of diagnosis of the patients were 5.56 ± 3.60 and 7.63 ± 3.65, respectively. The mean ages of the FMF patients and controls were 12 ± 4 and 10 ± 4, respectively. Routine laboratory parameters of FMF patients and healthy controls are presented in Table 1. No significant difference was observed between groups in CRP, WBC, platelet count, or most erythrocyte indices. However, several laboratory parameters differed significantly between FMF patients and controls. FMF patients showed higher AST, ALT, and ESR levels compared with controls (*p* < 0.05 for all). Hemoglobin, MCH, and MCHC levels were significantly lower in the FMF group, while RDW values were higher (*p* < 0.001). In leukocyte subgroups, absolute lymphocyte counts and lymphocyte percentages were significantly increased in FMF patients, whereas basophil and eosinophil percentages were significantly lower compared with controls.

Overall, these findings indicate the presence of mild alterations in hematological and inflammatory parameters in FMF patients despite comparable CRP levels between groups.

### 3.2. The Mutation Genotype Distribution of FMF Patients

The mutation analysis results of all 48 patients were obtained from their clinical records. Patients were classified according to the presence or absence of mutations involving exon 10 of the *MEFV* gene.

Exon 10 mutations were identified in 39 patients (81.3%), whereas 9 patients (18.7%) carried mutations located outside exon 10. Exon 10 involvement included homozygous, heterozygous, and compound heterozygous genotypes containing at least one exon 10 variant. All detected *MEFV* variants were systematically reviewed and classified at the variant level. For each patient, mutation type, zygosity, and exon location were recorded. Variant pathogenicity was evaluated using the INFEVERS database and curated mutation resources. To account for genetic heterogeneity and differences in variant penetrance, detailed variant-level information was compiled and summarized. A comprehensive overview of all identified *MEFV* variants, including zygosity, exon location, and pathogenic classification, is provided in Table 2. Due to the limited number of patients in some mutation subgroups, particularly those carrying less frequent variants such as V726A, subgroup analyses should be considered exploratory and interpreted with caution.

### 3.3. Relative Expression Levels of miR-223-3p and miR-204-3p Between Groups

**Figure 1:**
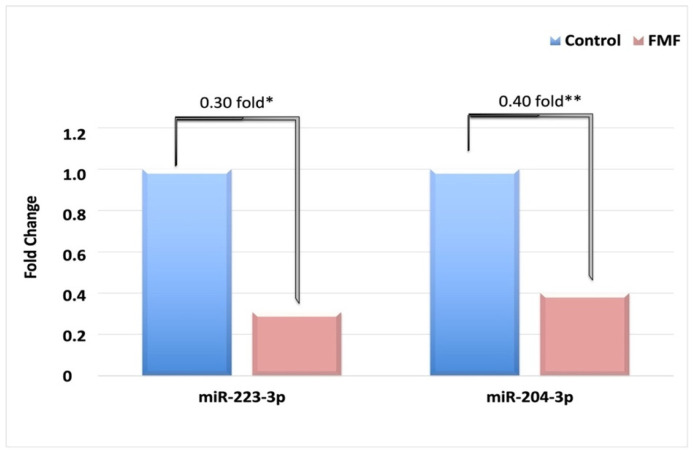
Relative expression levels of miR-223-3p and miR-204-3p between FMF patients and controls. miR-223-3p was found to be 0.30-fold downregulated, and miR-204-3p was found to be 0.40-fold downregulated in patients with FMF. * means *p* < 0.05, ** means *p* < 0.001.

The biomarkers we examined in the study are summarized in Table 3 and Figure 1. Accordingly, pyrin levels were found to be significantly higher in the FMF group (*p* < 0.001), while DTX1 levels were decreased in the FMF group (*p* = 0.036). No significant changes were found in CTLA-4 levels (Table 2).

As shown in Figure 1, miR-223-3p was found to be 0.30-fold downregulated, and miR-204-3p was found to be 0.40-fold downregulated in patients with FMF compared to the control group. FMF patients were also divided into subgroups according to their mutations.

**Figure 2:**
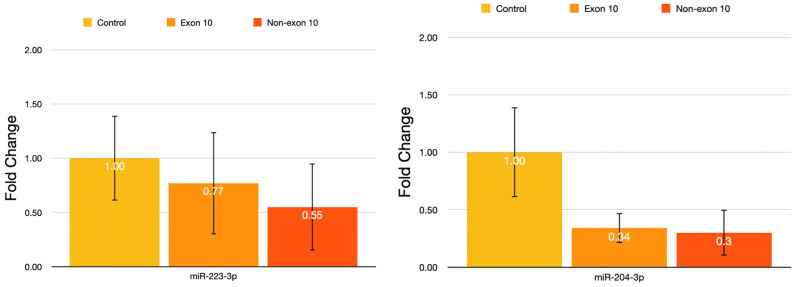
Relative expression levels of miR-223-3p and miR-204-3p according to exon 10 involvement and controls. FMF patients were categorized based on the presence or absence of exon 10 mutations in the MEFV gene. Expression levels were compared between exon 10-positive patients, non-exon 10 patients, and healthy controls.

As shown in Figure 2, miR-223-3p and miR-204-3p expression levels were evaluated according to the presence or absence of exon 10 mutations in FMF patients and compared with the control group. Mean miR-223-3p expression levels were lower in both FMF subgroups than in controls; however, pairwise comparisons revealed no statistically significant differences (control vs. exon 10, *p* = 0.659; control vs. non-exon 10, *p* = 1.000; exon 10 vs. non-exon 10, *p* = 0.835). Similarly, miR-204-3p expression levels were reduced in both exon 10-positive and non-exon 10 patient groups compared with controls, but these differences were not statistically significant (control vs. exon 10, *p* = 0.950; control vs. non-exon 10, *p* = 0.991; exon 10 vs. non-exon 10, *p* = 0.998). Overall, no statistically significant differences in miR-223-3p or miR-204-3p expression were observed among the groups.

Serum levels of pyrin, CTLA-4, and DTX1 were also compared among exon 10-positive patients, non-exon 10 patients, and controls (Table 4). A post-hoc power analysis demonstrated a statistical power of 99.2% for the comparison of pyrin biomarker levels between study groups. Mean pyrin levels showed a trend toward higher values in the non-exon 10 group; however, the difference was not statistically significant (*p* = 0.073). CTLA-4 levels were comparable across all groups (*p* = 0.682). Similarly, DTX1 levels did not differ significantly between groups (*p* = 0.096).

Overall, genotype stratification based on exon 10 involvement did not reveal statistically significant differences in miRNA expression or protein levels between FMF subgroups and controls.

**Figure 3:**
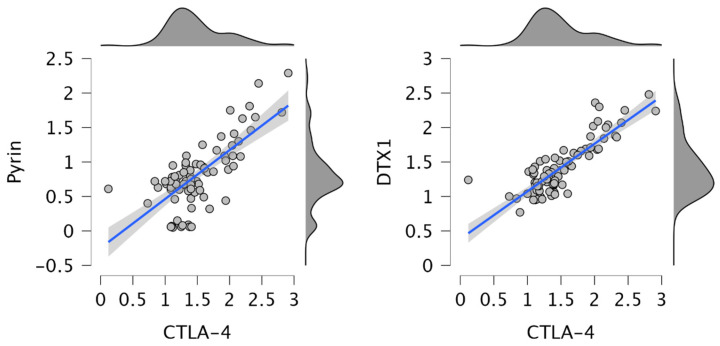
Correlations of CTLA-4 with pyrin and DTX1 levels.

Figure 3 shows that CTLA-4 levels were positively correlated with pyrin and DTX1 levels (r = 0.602; *p* < 0.001; r = 0.740; *p* < 0.001, respectively).

## 4. Discussion

FMF is caused by mutations in the *MEFV* gene, which encodes a pyrin protein consisting of 10 exons and 781 amino acids. Although more than fifty mutations have been identified to date, most of these mutations are rare. The most common mutations seen in FMF patients in our study were M694V/R202Q (33.33%; n = 16), M694V (27.08%; n = 13), and V726A (6.25%; n = 3). Our findings are consistent with previous reports, confirming M694V as the most prevalent FMF-associated mutation, accounting for approximately 20–80% of cases in the FMF patient population [10,11,12]. Although it is known which gene mutations cause the disease, the mechanisms by which this defect causes inflammatory attacks are largely unclear.

In the context of endotoxin-induced activation of mononuclear phagocytes, pyrin can enhance IL-1β processing and release. In our study, plasma pyrin levels were found to be significantly higher in children with FMF compared to controls. However, no significant difference was found between pyrin levels in the subgroups formed according to the most frequent mutations. This finding aligns with the basic pathogenesis of the disease and the role of the innate immune system.

Although activation of the innate immune system has been reported in FMF, information on adaptive system dysfunction in this disease is limited [13]. CTLA-4, also known as the first immune checkpoint CD152 in T cell activation, has been implicated in many autoimmune diseases [14,15,16]. Previous studies have shown that Th1 cell polarization can occur in FMF, and this may indicate that the adaptive immune response is involved in FMF [17,18]. However, in this study, no significant difference was found when plasma CTLA-4 levels were compared between groups. Gunesacar et al. [19] reported no association between the +49A/G polymorphism in the *CTLA-4* gene and FMF but found that the -318C/T polymorphism on the same gene may be associated with the non-autoimmune pathogenesis of the disease. Previous studies have also reported that the -318 T allele is associated with high promoter activity of the *CTLA-4* gene; in this context, the more common CT genotype and the presence of the T allele in FMF patients may partially explain the lack of autoimmune manifestations such as antigen-specific T cell activation and autoantibodies in this autoinflammatory disease. Interestingly, an increase in serum CTLA-4 was found in patients with autoimmune diseases compared to not only healthy donors but also those with non-autoimmune diseases [20,21]. The lack of change in plasma CTLA-4 levels in our study suggests that systemic inflammation occurs in FMF without T cell activation.

DTX1 is a ubiquitin E3 ligase containing a proline-rich motif. It is reported that DTX1 inhibits T cell activation by E3 ligase-dependent and independent mechanisms. It has been established in experimental studies that DTX1 deletion leads to hyperactivation of T cells and lupus-like autoimmune syndromes, but its relationship with human autoimmune diseases is not precisely known. Studies have suggested that it may be a negative regulator of T cell function. It has been suggested that it exerts this effect by increasing the production of IFN-γ [22]. According to the literature review, our study is the first to investigate the role of serum DTX1 in FMF. In our study, plasma DTX1 levels of FMF patients were found to be significantly lower compared to the control group. Although the exact pathogenesis of FMF is still unknown, our results demonstrate an association between circulating DTX1 levels and FMF status. The decrease in DTX1 levels may be due to IFN-γ. DTX1 may be involved in the regulation of FMF disease activity and may be a potential therapeutic agent. In addition, the positive correlation between DTX1 and CTLA-4 is one of our important findings. Although patients are usually asymptomatic between attacks, it is known that subclinical inflammation can continue. An event that normally causes mild inflammation can cause a severe inflammatory response in FMF patients. Although reduced DTX1 levels and the positive correlation between DTX1 and CTLA-4 may be consistent with persistent immune dysregulation during attack-free periods, these observations should be interpreted cautiously. In the absence of genotype-specific analyses and direct biomarker–phenotype correlations, DTX1 and CTLA-4 cannot be considered definitive markers of subclinical inflammation in this study. Rather, our findings are associative and hypothesis-generating, warranting validation in larger, genotype-stratified cohorts with longitudinal clinical follow-up. DTX1 and CTLA-4 levels are noninvasive, simple, and inexpensive parameters that reflect subclinical inflammation in FMF. In the future, these parameters may be important in detecting subclinical inflammation in patients with FMF and preventing devastating complications of FMF (such as amyloidosis).

Variants such as R202Q and E148Q, which are generally regarded as variants of uncertain clinical significance or low penetrance, were evaluated separately from classical exon 10 mutations. Although these variants are not consistently recognized as independently disease-causing, accumulating evidence suggests that their frequent occurrence in compound heterozygous states with pathogenic exon 10 mutations may indicate a potential modifier role in disease expression [23,24]. Accordingly, their inclusion reflects the genetic heterogeneity commonly observed in pediatric FMF cohorts and enables investigation of epigenetic and inflammatory signatures beyond strict genotype–phenotype associations. Recent studies examining the clinical relevance of the E148Q and R202Q variants further support consideration of their possible contribution to phenotypic variability [23,24]. Some patients were found to have negative MEFV mutations, and these patients were reported to have a lower rate of late-onset FMF patients with a family history of FMF. However, the definitive presence of patients without such mutations suggests the existence of additional causes for disease development, including mutation in alternative genes and possibly the emergence of epigenetic dysregulation [25,26]. The identification of these epigenetic changes is important for patient diagnosis and follow-up. Among the epigenetic mechanisms, the effects of miRNAs have come to the fore in recent years. Dysregulation of different miRNAs has been demonstrated in FMF patients, and against inflammatory stimuli, miRNAs have been shown to regulate innate immune responses. Hortu et al. showed that 11 miRNAs (miR-125a, -132, -146a, -155, -15a, -16, -181a, -21, -223, -26a, and -34a) revealed that their expression levels were significantly lower [27]. Wada et al. [7] reported that the expression patterns of circulating miRNAs differed between FMF subgroups based on MEFV mutations between FMF episodes. However, in our study, no effect of different mutations on miRNA expression levels was found.

The miRNA profiling of circulating CD4+ Tregs in adults has been confirmed in several miRNAs, including miR-223, to regulate Foxp3 and CTLA-4 expression [28]. miR-223 is an intrinsic modulator of neutrophil sensitivity, similar to the proposed role for miR-181 acting as a “rheostat” controlling T cell activation [29]. In this context, we aimed to study the CTLA-4 molecule, which we think is associated with the pathogenesis of FMF, and the miR-223-3p and DTX1 molecules targeting the gene encoding this molecule, and the miR-204-3p targeting the coding gene. It was decided to study miRNA-204-3p, which we determined to target the DTX1 gene because of the database review. In the study, according to the calculated fold change, miR-204-3p and miR-223-3p levels were found to be statistically significantly decreased in the FMF patient group compared to the healthy control group. However, no correlation was found with other parameters. These results support the role of miRNAs in the etiopathogenesis of FMF. Koga et al. [30] reported that serum miR-204-3p levels in FMF patients may have the potential to be a useful biomarker. They also showed that miR-204-3p targeted the PI3Kg pathway, inhibiting the production of the inflammatory cytokine (IL-6) in FMF. Hortu et al. [27] reported that the expression of miR-223 was lower in the FMF patient group than in the control group. These findings support our conclusion and show that miR-223 may be effective in the pathogenesis of FMF. miR-223 is thought to have a key role in the regulation of NLRP3 in neutrophils. Downregulation of miR-223 by IL-6 has been reported to promote IL-1β and IL-6 production [31]. Among our findings, the decrease in miR-223 expression in patients with FMF confirms these findings. Although miRNAs associated with inflammation have been identified in the systemic circulation, their roles and underlying mechanisms in individuals with FMF have not yet been clarified. Prospective clinical studies should be conducted to determine the utility of serum miR-204-3p in detecting FMF and assessing disease activity, as well as the sensitivity and specificity of its measurement.

### 4.1. Clinical Relevance

The identification of altered plasma pyrin and DTX1 levels, together with dysregulated miR-204-3p and miR-223-3p expression, provides clinically relevant insight into the persistence of subclinical inflammation in FMF, even during attack-free periods. These molecular signatures may contribute to improved risk stratification, disease monitoring, and early detection of ongoing inflammatory activity in FMF patients. DTX1 may represent a promising biomarker for assessing quiescent-phase inflammatory burden, while microRNA profiling could support the development of personalized follow-up strategies and therapeutic decision-making, including the identification of patients at increased risk for amyloidosis or colchicine resistance.

### 4.2. Study Limitations and Strengths

The most important limitation of our study is the relatively small number of patients and the absence of a patient group during the attack period. In addition, the case–control design precludes causal inference; therefore, all findings should be interpreted as associative. Genotype–biomarker and biomarker–phenotype relationships may also be influenced by residual confounding, selection bias, and reverse causation. Another limitation of this study is the relatively small size of certain mutation subgroups and the use of targeted mutation panels rather than full MEFV gene sequencing in all patients. Additionally, variants with uncertain or low penetrance, such as R202Q, were interpreted cautiously, particularly when present in combination with pathogenic exon 10 mutations. On the other hand, the investigation of plasma pyrin and DTX1 levels in our study and the absence of a similar study in the literature make our study unique. Nevertheless, given the genetic heterogeneity of FMF and the limited size of mutation-specific subgroups, the present findings should be interpreted as hypothesis-generating rather than definitive, warranting validation in larger, genotype-stratified cohorts. Although controls were matched for age, a difference in sex distribution between the FMF and control groups was present, which may have introduced residual confounding and should be considered when interpreting the results. Another limitation is the lack of systematic hemolysis assessment, which may affect circulating miRNA measurements due to the release of erythrocyte-derived miRNAs into serum. Another limitation of this study is the use of RNU6 (U6) as the endogenous control for serum miRNA normalization, as U6 expression may be unstable in circulating samples and influenced by disease state or pre-analytical variability, potentially affecting between-group comparisons. Another limitation is that pyrin and DTX1 are primarily intracellular proteins, and the precise biological source of their detectable plasma levels (e.g., soluble forms, extracellular vesicle-associated proteins, or release due to cellular turnover) could not be determined. CTLA-4 was measured in plasma using an ELISA detecting circulating (soluble) CTLA-4, which does not directly reflect membrane-bound CTLA-4 expression on T cells, limiting the biological interpretation of these findings.

### 4.3. Conclusions

Our findings indicate that plasma pyrin levels are increased in FMF and may be associated with heightened inflammasome-related inflammatory activity. Lower DTX1 levels and the positive correlation between DTX1 and CTLA-4 were also observed in FMF patients during attack-free periods, suggesting a potential association with ongoing immune dysregulation. In this context, DTX1 may represent a candidate biomarker for monitoring inflammatory burden in the quiescent phase. Additionally, the reduced expression of miR-204-3p and miR-223-3p in FMF is consistent with prior reports and may be linked to proinflammatory cytokine signaling, supporting their potential involvement in FMF-related inflammatory pathways. Given the case–control design and the absence of genotype-specific and clinical correlation analyses, these observations should be considered preliminary and require validation in larger, genotype-stratified, longitudinal studies. The proposed miR-223-3p/CTLA-4 and miR-204-3p/DTX1 relationships are based on target prediction databases and prior literature and should not be interpreted as evidence of direct regulatory interactions in the present study.

### 4.4. Future Research

Further large-scale, multicenter studies incorporating patients evaluated during both attack and remission phases are warranted to validate the present findings. Future research should also explore the potential roles of pyrin, DTX1, CTLA-4, miR-204-3p, and miR-223-3p in key clinical outcomes of FMF, including early disease susceptibility, disease severity, risk of amyloidosis, and resistance to colchicine therapy. Longitudinal studies may help clarify their utility as diagnostic, prognostic, or therapeutic biomarkers in FMF. Future studies should therefore incorporate validated circulating reference miRNAs and/or exogenous spike-in controls to improve normalization, accuracy, and robustness.

## Figures and Tables

**Table 1:** Routine laboratory findings of FMF patients and healthy controls.

Parameter	Control (Mean ± SD)	FMF (Mean ± SD)	*p* Value
CRP (mg/dL)	1.20 ± 1.44	1.35 ± 1.67	0.658
AST (U/L)	20.85 ± 5.24	25.69 ± 13.03	0.023
ALT (U/L)	12.56 ± 3.95	19.63 ± 18.71	0.014
ESR (mm/h)	4.69 ± 1.81	11.23 ± 11.93	<0.001
WBC (×10^3^/µL)	7.12 ± 1.00	7.10 ± 1.41	0.934
RBC (M/µL)	4.92 ± 0.28	4.81 ± 0.34	0.122
Hemoglobin (g/dL)	13.96 ± 0.99	12.83 ± 1.27	<0.001
Hematocrit (%)	39.29 ± 2.35	38.21 ± 3.50	0.100
MCV (fL)	79.81 ± 2.64	79.28 ± 5.68	0.568
MCH (pg)	28.30 ± 1.15	26.68 ± 1.98	<0.001
MCHC (g/dL)	35.47 ± 0.73	33.50 ± 0.68	<0.001
RDW (%)	13.63 ± 0.55	14.57 ± 1.64	<0.001
Platelet (K/µL)	283 ± 46	265 ± 59	0.125
MPV (fL)	8.80 ± 0.72	8.72 ± 0.92	0.671
PCT (µg/L)	0.24 ± 0.04	0.22 ± 0.05	0.178
PDW (fL)	16.62 ± 0.39	16.58 ± 0.51	0.673
Neutrophils (K/µL)	3.59 ± 0.77	3.81 ± 1.47	0.369
Lymphocytes (K/µL)	2.83 ± 0.44	3.22 ± 0.91	0.012
Monocytes (K/µL)	0.56 ± 0.06	0.53 ± 0.15	0.192
Eosinophils (K/µL)	0.21 ± 0.11	0.17 ± 0.17	0.208
Basophils (K/µL)	1.69 ± 1.62	0.19 ± 0.85	<0.001
Neutrophils (%)	48.84 ± 4.19	48.06 ± 11.85	0.674
Lymphocytes (%)	38.28 ± 4.16	42.74 ± 10.60	0.010
Monocytes (%)	8.23 ± 1.01	7.80 ± 1.96	0.197
Eosinophils (%)	4.95 ± 1.77	2.43 ± 2.29	<0.001
Basophils (%)	1.11 ± 1.01	0.52 ± 0.24	0.001

Abbreviations: CRP, C-reactive protein; AST, aspartate aminotransferase; ALT, alanine aminotransferase; ESR, erythrocyte sedimentation rate; WBC, white blood cell count; RBC, red blood cell count; MCV, mean corpuscular volume; MCH, mean corpuscular hemoglobin; MCHC, mean corpuscular hemoglobin concentration; RDW, red cell distribution width; MPV, mean platelet volume; PCT, plateletcrit; PDW, platelet distribution width.

**Table 2:** Detailed distribution and classification of MEFV variants in FMF patients.

*MEFV* Variant	Zygosity	Exon Location	Pathogenic Classification *	*n*
M694V	Homozygous	Exon 10	Pathogenic	3
M694V	Heterozygous	Exon 10	Pathogenic	10
M694V/R202Q	Compound heterozygous	Exon 10/Exon 2	Pathogenic/VUS	16
V726A	Homozygous	Exon 10	Pathogenic	3
M680I	Heterozygous	Exon 10	Pathogenic	1
M680I/V720A	Compound heterozygous	Exon 10/Exon 10	Pathogenic/Likely pathogenic	1
M680I/V726A	Compound heterozygous	Exon 10/Exon 10	Pathogenic	2
M694V/V726A	Compound heterozygous	Exon 10/Exon 10	Pathogenic	1
V726A/R202Q	Compound heterozygous	Exon 10/Exon 2	Pathogenic/VUS	1
K695R	Heterozygous	Exon 10	Likely pathogenic	1
E148Q/N	Heterozygous	Exon 2	VUS	2
E148Q/R202Q	Compound heterozygous	Exon 2/Exon 2	VUS	1
R202Q	Heterozygous	Exon 2	VUS/Low penetrance	6

* Pathogenic classification based on the INFEVERS database and curated mutation databases. Abbreviations: VUS, variant of uncertain significance.

**Table 3:** Inflammation markers between the control and FMF groups.

	Control (*n* = 36) Mean ± S.D. (Median, Lower-Upper Range)	FMF (*n* = 48) Mean ± S.D. (Median, Lower-Upper Range)	*p*
Pyrin (ng/L)	0.61 ± 0.35 (0.61, 0.06–1.75)	0.97 ± 0.40 (0.78, 0.05–2.29)	0.001
CTLA-4 (ng/mL)	1.49 ± 0.50 (1.40, 0.12–2.81)	1.49 ± 0.44 (1.37, 0.73–2.91)	0.964
DTX1 (ng/mL)	1.51 ± 0.41 (1.42, 0.77–2.48)	1.34 ± 0.34 (1.24, 0.95–2.25)	0.036
CTLA-4, cytotoxic T-lymphocyte-associated antigen gene-4; DTX1, deltex 1.			

**Table 4:** Pyrin, CTLA-4, and DTX1 levels according to exon 10 involvement and the control group.

	Subgroups	Mean ± Std. Deviation	*p* Value
Pyrin (ng/L)	Control	0.702 ± 0.447	0.073
	Exon 10	0.854 ± 0.407	
	Non-exon 10	1.062 ± 0.588	
CTLA-4 (ng/mL)	Control	1.493 ± 0.500	0.682
	Exon 10	1.460 ± 0.393	
	Non-exon 10	1.612 ± 0.634	
DTX1 (ng/mL)	Control	1.512 ± 0.408	0.096
	Exon 10	1.323 ± 0.323	
	Non-exon 10	1.403 ± 0.426	

## Data Availability

The datasets used and/or analyzed during the current study are available from the corresponding author on reasonable request.
